# Supplementary material for: Parallel nonlinear neuromorphic computing with temporal encoding
Source: Sci Adv. 2026 Jul 29;12(31):eaea1114. doi: 10.1126/sciadv.aea1114 (PMC13418742; doi:10.1126/sciadv.aea1114)
Supplement: Supplementary file 1 — Supplementary notes S1 to S9 Figs. S1 to S10 References [file sciadv.aea1114_sm.pdf]

Science Advances
AAAS

Supplementary Materials for

Parallel nonlinear neuromorphic computing with temporal encoding

Guangfeng You *et al.*

Corresponding author: Chao Qian, chaoq@intl.zju.edu.cn; Hongsheng Chen, hansomchen@zju.edu.cn

Sci. Adv. **12**, eaea1114 (2026)
DOI: 10.1126/sciadv.aea1114

This PDF file includes:

Supplementary notes S1 to S9
Figs. S1 to S10
References

Supplementary Note 1: Governing equations for metasurface layout and Temporal encoding nonlinearity in far field propagation

Metasurface layouts manipulate wave scattering through controlling the response function t. Specifically, the corresponding governing equations or manipulating mechanism of metasurface layouts are,

$$y_i^{M+1} = \left|m_i^{M+1}\right|^2 \tag{S1}$$

$$m_i^l = \sum_k n_{k,i}^{l-1} = w_{k,i}^{l-1} \cdot t_k^{l-1} \cdot m_k^{l-1} \tag{S2}$$

$$t_i^l = \sum_{p=1}^{P} a_{i,p}^l \cdot e^{j\phi_{i,p}^l} \tag{S3}$$

t_i^l is the response function of i_{th} metasurface unit cell at l_{th} layer, manipulated by temporal sequence $\boldsymbol{S} = [\boldsymbol{D}_1, \boldsymbol{W}_2, \boldsymbol{D}_3, \boldsymbol{W}_4, \dots, \boldsymbol{D}_{N-1}, \boldsymbol{W}_N]$, where $a_{i,p}^l$ and $\phi_{i,p}^l$ are the amplitude and phase of i_{th} metasurface unit cell at $\boldsymbol{W}_p$ or $\boldsymbol{D}_p$. P is determined by temporal sequence length. By manipulating the amplitude $a_{i,p}^l$ or phase $\phi_{i,p}^l$, one can control the system response at output plane. y_i^{M+1} is the intensity of the i_{th} resulting optical field detected by output plane. m_i^l is the input wave to i_{th} metasurface unit cell and $n_{k,i}^{l-1}$ is output function of k_{th} metasurface unit cell at $(l-1)_{th}$ layer, illuminating to the i_{th} metasurface unit cell at l_{th} layer. $w_{k,i}^{l-1}$ is the propagation functions between k_{th} metasurface unit cell at $(l-1)_{th}$ layer and i_{th} metasurface unit cell at l_{th} layer. In classification task, we record responses of four receiver area at output plane for multi-label classification, and ten receiver area at output plane for *MNIST, Fashion-MNIST, and CIFAR-10* classification. All the variables above are working at $f_c + n\Delta f$, where f_c is the incident frequency and Δf is the modulating frequency of the temporal sequence.

Both frequency-modulation scheme for parallel task and the dynamic-memory mechanism for maze-solving are achieved by controlling the response function t through optimizing metasurface layout. By optimizing metasurface layouts, the accuracy of parallel tasks and memory capacity of reinforcement agent for maze-solving are iteratively improved. The divergent requirements or difference between "multi-task parallelism" and "maze solving" is the additional introduction of asynchronous modulating mechanism for implementation of parallel tasks while the maze-solving problem working in a single modulating frequency. Specifically, for "multi-task parallelism", Eq. (S1) is extended to asynchronous form $t_i^{l,f_c+\Delta f_t} = \sum_{p=1}^{P} a_{i,p}^{l,f_c+\Delta f_t} \cdot e^{j\phi_{i,p}^{l,f_c+\Delta f_t}}$, where t_i^l is the response function of i_{th} metasurface unit cell at l_{th} layer working at asynchronous modulating frequency $f_c + \Delta f_t$. The corresponding Eqs. (S2) and (S3) only involve the response functions $t_i^{l,f_c+\Delta f_t}$ working at

respective asynchronous modulating frequency, i.e., $m_i^{l,f_c+\Delta f_1} = \sum_k n_{k,i}^{l-1,f_c+\Delta f_1} = w_{k,i}^{l-1,f_c+\Delta f_1} \cdot t_k^{l-1,f_c+\Delta f_1} \cdot m_k^{l-1,f_c+\Delta f_1}$ for metasurface layouts working at asynchronous modulating frequency $\Delta f_t = \Delta f_1$, and $m_i^{l,f_c+\Delta f_2} = \sum_k n_{k,i}^{l-1,f_c+\Delta f_2} = w_{k,i}^{l-1,f_c+\Delta f_2} \cdot t_k^{l-1,f_c+\Delta f_2} \cdot m_k^{l-1,f_c+\Delta f_2}$ for metasurface layouts working at asynchronous modulating frequency $\Delta f_t = \Delta f_2$.

Next, we illustrate that the proposed temporal encoding nonlinearity is also applicable in far field (63). Similarly, given the source data $\boldsymbol{D}$ and weight matrices $\boldsymbol{W}$, the propagation function at far field region is:

$$F(\theta,\varphi,t) = \sum_p \sum_q E_{pq}(\theta,\varphi)\,\Gamma_{pq}(t)e^{j\frac{2\pi}{\lambda_c}[(p-1)dxsin\theta cos\varphi+(q-1)dysin\theta sin\varphi]} \qquad \text{(S4)}$$

where $E_{pq}(\theta,\varphi)$ is the far-field pattern at (p,q) metasurface unit cell. θ and φ are elevation and azimuth angles, respectively. Following the Fourier expansion in the main text, the stable propagation function at frequency $f_c + m\Delta f_0$ is:

$$F(\theta,\varphi,t) = \frac{1}{L}\sum_p \sum_q \sum_l E_{pq}(\theta,\varphi)\Gamma_{pq}^l sinc(\frac{\pi m}{L})e^{-j\frac{\pi m(2n-1)}{L}}e^{j\frac{2\pi}{\lambda_c}[(p-1)d_x sin\theta cos\varphi+(q-1)d_y sin\theta sin\varphi]} \qquad \text{(S5)}$$

where L is length of the time-varying sequence. Γ_{pq}^l is given by

$$\boldsymbol{\Gamma}^l = \begin{cases} \boldsymbol{W}_l, & l = 2i \\ \boldsymbol{D}_l, & x = 2i+1 \end{cases} \qquad \text{(S6)}$$

where i is a non-negative integer. $\boldsymbol{D}_l$ and $\boldsymbol{W}_l$ denote the source data and weight matrix at l_{th} time partition. Using multiple scattering between linear metasurfaces, we construct a nonlinear mapping between data and system response, which can be simplified into the form of $\boldsymbol{f}(\boldsymbol{D},\boldsymbol{W}) = (\boldsymbol{a}\cdot\boldsymbol{D}^3 + \boldsymbol{b}\cdot\boldsymbol{D}^2 + \boldsymbol{c}\cdot\boldsymbol{D} + \boldsymbol{d})$ for a system composed of three metasurfaces. a, b, c, and d are related with system configuration. This nonlinearity achieved with far-field multiple scattering provides an efficient tool for future communication systems.

Supplementary Note 2: Linear degradation induced by spatial data-repetition strategy

As shown in Fig. S4a, for a diffraction neural network operator with a trainable diffraction layer $t(x',y')$, the relation between input $i(x'',y'')$ and output aperture $O(x,y)$ is:

$$O(x,y) = \sum_{x',y'}\sum_{x'',y''} E\big(i(x'',y'')\big)\cdot G_1(x'-x'',y'-y'')\cdot t(x',y')\cdot G_2(x-x',y-y')$$

$$= \sum_{x'',y''} E\big(i(x'',y'')\big)\cdot \sum_{x',y'} G_1(x'-x'',y'-y'')\cdot t(x',y')\cdot G_2(x-x',y-y')$$

$$= \sum_{x'',y''} E(i(x'',y'')) \cdot h(x'',y'',x,y) \tag{S7}$$

where $G_1(x'-x'',y'-y'')$ and $G_2(x-x'',y-y'')$ are the space propagation functions. E is the data encoding function of the input. The term $h(x'',y'',x,y) = \sum_{x',y'} G_1(x'-x'',y'-y'') \cdot t(x',y') \cdot G_2(x-x',y-y')$ is the corresponding PSF. By optimizing $t(x',y')$, any arbitrary complex-valued linear transformation can be realized, which is also the requirement of linear expressivity in neural network.

However, as shown in Fig. S4b, when the data-repetition strategy is introduced to achieve nonlinearity where the input $i(x',y')$ is repeated on the diffraction layer through certain encoding function $f(i(x',y'))$, the relation between $i(x'',y'')$ and $O(x,y)$ becomes:

$$O(x,y) = \sum_{x',y'} \sum_{x'',y''} E\big(i(x'',y'')\big) \cdot G_1(x'-x'',y'-y'') \cdot t(x',y') \cdot f\big(i(x',y')\big) \cdot G_2(x-x',y-y')$$

$$= \sum_{x'',y''} E(i(x'',y'')) \cdot \sum_{x',y'} G_1(x'-x'',y'-y'') \cdot t(x',y') \cdot f(i(x',y')) \cdot G_2(x-x',y-y') \tag{S8}$$

Then, the PSF term becomes input-dependent:

$$h'(x'',y'',x,y) = \sum_{x',y'} G_1(x'-x'',y'-y'') \cdot t(x',y') \cdot f(i(x',y')) \cdot G_2(x-x',y-y') \tag{S9}$$

From Eq. (S9), the linear transformation (i.e., linear expressivity) is diminished or eliminated by additional introduction of input data $i(x',y')$ on $t(x',y')$. It means that such input-dependent spatially-varying PSF can no longer perform arbitrary complex-valued linear transformation. Therefore, such data-repetition diffractive structures cannot perform arbitrarily selected fully connected or convolutional layers commonly used in artificial neural networks.

Assuming encoding function being $f\big(i(x',y')\big) = \phi_n = i(x',y') \odot s_{Ln} + b_{Ln}$, where ϕ_n is the phase and $\odot$ represents the element-wise multiplication. s_{Ln} and b_{Ln} are the trainable scaling and bias parameters. In the extreme condition of $s_{Ln} = 0$, $f\big(i(x',y')\big)$ will turns into a constant bias matrix, and the whole diffraction network operator is transformed into classical linear diffraction operator. However, this operation inevitably results in the disappearance of the original nonlinearity.

Supplementary Note 3: Backward propagation of the temporal neural network

The backpropagation algorithm is utilized to train the temporal neural network using stochastic gradient descent optimization. The optimization objective is to minimize the loss function, which is formulated as modified cross-entropy loss function:

$$L = -\frac{1}{N}\sum_{i=1}^{N}\sum_{c=1}^{K} y_{ic} \cdot \log(f(h_\theta(x_{ic}))) + \alpha \cdot D(F) \quad \text{(S10)}$$

where x_{ic} is the i_{th} input sample corresponding to category y_{ic}. $s_i^{M+1} = f(h_\theta(x_{ic}))$ is the output intensity obtained by receiver detector. $u_i^{M+1} = h_\theta(x_{ic})$ denotes output of the temporal neural network with trainable parameter θ (i.e., trainable weight matrices). Based on soft-argmax function, the network identifies every weight element's modification as a probability update event, which maps discrete weight value into a range of continuous probability value. For instance, presuming that each reconfigurable metasurface unit possesses merely two states (0 and 1), the Bernoulli distribution maybe employed to characterize each state transition event. That is, the probability of the unit residing in state 0 is denoted as ρ, while the probability of state 1 is represented as $1-\rho$. Consequently, we can employ the update probability methodology to facilitate the training of the network effectively. α is correct factor. $D(F) = \sum_t^T \sum_l^L F(p_i^l) \cdot (1 - F(p_i^l))$ denotes correction term for the probability-based strategy, which makes the soft-argmax approximation $F(p_i) = \frac{e^{\beta p_i}}{\sum_k e^{\beta p_k}}$ of continuous phase states approach the discrete binary states. L and T denote the number of neurons in each layer and number of layers, respectively. Following the propagation function in Eq. (2), the gradient of L to trainable parameter θ_i (e.g. amplitude or phase of unit cell of the $\boldsymbol{W}$) in l_{th} time partition at layer t is expressed as:

$$\frac{\partial L}{\partial \theta_{l,i}^t} = \frac{1}{K}\sum_k (s_k^{T+1} - y_k)\, real\left\{(u_k^{T+1})^* \cdot \frac{\partial u_k^{T+1}}{\partial \theta_{l,i}^t} \cdot e^{-i2\pi lm\Delta f_0 \tau}\right\} + \frac{\partial D}{\partial \theta_{l,i}^t} \quad \text{(S11)}$$

Computation of $\frac{\partial u_k^{T+1}}{\partial \theta_{l,i}^t}$ can be implemented with chain rules (20). For the layer $t = T$,

$$\frac{\partial u_k^{T+1}(x_k, y_k, z_k)}{\partial \theta_{l,i}^{t=T}} = j \cdot \theta_{l,i}^T(x_i, y_i, z_i) \cdot u_i^T(x_i, y_i, z_i) \cdot w_i^T(x_k, y_k, z_k) \quad \text{(S12)}$$

where $w_i^T(x_k, y_k, z_k)$ is propagating operator in free space. Similarly, $\frac{\partial u}{\partial \theta}$ at layer $t = T - q$ is $\frac{\partial u_k^{T+1}}{\partial \theta_{l,i}^{t=T-q}} = j \cdot \theta_{l,i}^{T-q} \cdot u_i^{T-q} \cdot \sum_{k_1} w_{k_1}^T \cdot \theta_{l,k_1}^T \cdots \sum_{k_m} w_{k_m}^{T-q+m} \cdot \theta_{l,k_m}^{T-q+m} \cdot w_{k_m}^{T-q}$. In the backpropagation, the parameters of temporal neural network are updated per iteration and the results of each round

of updates are converted into discrete states according to the probability-based strategy and participate in the next iteration.

Supplementary Note 4: Performance influence of linear model with finite input aperture

In the proposed framework, the information contained within the input data is not encoded through the input mask-based encoding of incident light, but is instead facilitated by the modulation of the metasurface. Consequently, the linear information processing capacity is governed by the encoding methodology employed. For convenience, we posit that our system uses the metasurfaces with the size of $M \times N$. In the fully connected operational mode, the input image is discretized into $M \times N$ matrix and integrated with corresponding weight matrices to synthesize a time-varying sequence of length L. In this case, we can draw a conclusion analogous to that of the conventional diffractive surface (64), that is, the dimension of the all-optical solution space of the linear diffractive network is linearly proportional to the length of the time-varying sequence that manipulates the diffractive surface. In the context of a single metasurface system, the upper bound of information processing capacity is fundamentally constrained by the spatial extent of the metasurface fields-of-view and output fields-of-view. In the convolution mode, the image with arbitrary size is partitioned into data matrices with the size of $M \times N$ and allocated across different temporal partitions of the time-varying sequence. At each time partition, our system functions as a quasi-static metasurface system, executing linear convolution operations between data and weight kernels via inter-metasurface scattering. In this scenario, the upper bound of information processing capacity is primarily determined by the extent of the output field-of-view. On the other hand, due to the inherent nonlinearity of proposed system, our metasurface processor exhibits theoretical proficiency in synthesizing novel output information from limited input data. This emergent nonlinear capability enables the reconstruction of comprehensive data characteristics through sufficient training, thereby transcending linear processing constraints.

Supplementary Note 5: Poisson dynamics during wave propagation

Here we investigate the relation between the input data and scattering field from a probabilistic standpoint. The input data is conceptualized as charge distribution on the $z = 0$ plane, migrating towards infinity along the electric field lines, eventually forming a uniform distribution on the hemisphere at infinity. The inverse process is referred as Poisson process, where negative charges are uniformly distributed on the hemisphere as the radius $r \to \infty$. These charges finally propagate along

the electric field lines to the $z = 0$ plane and exhibits data distribution, which can be represented by Poisson equation:

$$\nabla^2 \varphi(\boldsymbol{x}) = -\rho(\boldsymbol{x}) \tag{S13}$$

where $\rho(\boldsymbol{x})$ is source distribution and ∇^2 is Laplacian operator. $\varphi(x)$ is potential function and electric field $E(\boldsymbol{x}) = -\nabla\varphi(\boldsymbol{x})$. Thus, Eq. (S13) can be rewritten as $\nabla \cdot E = \rho$. The Eq. (S13) with zero boundary at infinity admits an integral solution $\varphi(\boldsymbol{x}) = \int G(\boldsymbol{x}, \boldsymbol{y})\, \rho(\boldsymbol{x}) d\boldsymbol{y}$, and $G(\boldsymbol{x}, \boldsymbol{y})$ is the Green function in the N dimensional space. According to the Green theorem, we get $E(\boldsymbol{x}) = -\nabla \mathrm{G}(\boldsymbol{x}, \boldsymbol{y})$ and $\varphi(\boldsymbol{x}) = \mathrm{G}(\boldsymbol{x}, \boldsymbol{y})$, when $\rho(\boldsymbol{x})$ is selected as point source. We can further define the data charge propagation process into a probabilistic flow model following Eq. (S13), which probability distribution of data $p_t(\boldsymbol{x})$ evolves according to the flow (65):

$$\frac{\partial p_t(x)}{\partial t} = -\nabla \cdot (p_t(\boldsymbol{x}) E(\boldsymbol{x})) \tag{S14}$$

The mechanism of Eq. (S13) is essentially consistent with the mechanism of the diffusion model (66), allowing us to explain its propagation process by perturbation (i.e., adding noise). Propagation process is decomposed into a series of time intervals and noise is added to the data distribution at each time step. The perturbed electric field may be regarded as the outcome of a weighted multiplication of the original electric field, reflecting the varying influences exerted by different regions of the data on the electric field. This process culminates in that after numerous time steps, the perturbed electric field enhances the areas with high-weight influences. This enables us to assess the impact of data areas on recognition performance by analyzing the intermediate results of the scattering process in the form of attention map, as shown in Fig. 3b. Moreover, we compare attention map at metasurface layer 1 and metasurface layer 2. As shown in Fig. S2, there exist subtle distinctions between attention maps at different layers and the attention maps at metasurface layer 2 capture complete information about labels of input data.

Supplementary Note 6: Dynamic memory capacity of metasurface agent

Maze-solving task implemented with reinforcement learning can essentially be reduced to a partially observable Markov decision process $\mathcal{M}_p(p) = \langle S, A, O, R, Z \rangle$, which is composed of maze states S, actions A, a set of observation O, observation function Z and reward R. An agent takes an action based on the observed history $h_{0:t-1} = \{o_i, a_i, r_i\}_{i=0:t-1}$ and receives a reward r_t at time t. To operate effectively in such Markov decision process, the agent must have memory mechanism to

retrieve a history $h_{0:t-1}$, which refers to the recording and storage of the observed states, actions taken, and rewards obtained by the agent during its interaction with the environment. The agent leverages the observations stored in its memory pool to engage in iterative learning and training, thereby enabling the vehicle to review its prior successful exploratory experiences (experience playback) at each location. For instance, as depicted in Fig. S3a, the unfinished trained metasurface agent offers appropriate guidance at the current position (3, 8), while delivering an erroneous policy at the position (4, 8). As the training progresses, the agent progressively retains the exploratory experiences from all positions, facilitating its ability to provide accurate directional guidance for the vehicle positioned at any given location (Fig. S3b).

Supplementary Note 7: Benchmark between proposed work and exiting neural networks in classification and reinforcement learning

As shown in Fig. S5 and Fig. S6, we first made comparisons with three representative datasets (i.e., *MNIST* for handwritten digit recognition, *CIFAR-10* and *Fashion-MNIST* for complex object recognition) by using our work, fully connected digital neural network (FCNN), nonlinear neural network implemented by spatial data-repetition strategy (27), and purely linear diffractive neural network (10). In the *MNIST* benchmark, as shown in Fig. S5a, both our framework and FCNN attained a recognition accuracy of over 90%. In the *CIFAR-10* and *Fashion-MNIST* benchmark experiments, as shown in Figs. S5b-c, it is evident that both FCNN and linear diffractive neural network underperform significantly compared to our method. This disparity mainly arises from the FCNN's inherent difficulty in extracting essential features from complex images. In contrast, our framework leverages more comprehensive feature extraction, as shown in Fig. 4a, to more effectively capture fundamental features and intricate details, thereby achieving superior performance.

All the models have the same structures, consisting of multiple-layers structures. The pixel size of metasurfaces used for the *MNIST* is set to 14 x 14 and 16 x 16 for *Fashion-MNIST* and *CIFAR-10*. The length of temporal sequences for the spatiotemporal metasurfaces is set to 8. For example, for the classification task based on *MNIST* dataset, the data $\boldsymbol{D}$ is partitioned into four feature maps $[\boldsymbol{D}_1, \boldsymbol{D}_2, \boldsymbol{D}_3, \boldsymbol{D}_4]$ and the complete temporal sequence for the first metasurface is $[\boldsymbol{D}_1, \boldsymbol{W}_1^1, \boldsymbol{D}_2, \boldsymbol{W}_2^1, \boldsymbol{D}_3, \boldsymbol{W}_3^1, \boldsymbol{D}_4, \boldsymbol{W}_4^1]$, where $\boldsymbol{W}_i^1$ is the weight matrices of first metasurface. The hidden size of FCNN is set to (28 x 28, 512, 256, 10) and the activation function is set to ReLU function. The hidden size of FCNN is (28 x 28, 512, 256, 10). The network implemented by our proposed method is manipulated by the temporal sequence with the size of (3,8,12,12). The batch size is set to 128. The

process of the three channel images in *CIFAR-10* for our framework is shown in Fig. R8, and the data in three channels are weighted summed and passed through threshold filters to generate binary data. The input for FCNN is flattened into size of (1,3 x 28 x 28).

For reinforcement learning, as shown in Fig. S6b, both our work and the nonlinear model implemented by spatial data-repetition exhibit better performance than purely linear model. The input data is modulated into an 11 x 11 maze matrix and a matrix representing the car's current position. This is a high-dimensional representation and does not contain complex image details, similar to the images in *CIFAR-10* dataset. Therefore, the network's performance relies more on the nonlinear extraction of high-dimensional information from the input for subsequent action selection. In contrast, purely linear system lacks the ability to extract high-dimensional features, resulting in a poor performance. The results in Fig. S7 demonstrate that our framework achieves better performance than previous data- repetition strategies implemented in purely spatial dimension. This outcome arises not only from the expansion of the trainable parameter space but, more critically, from our ability to realize efficient nonlinearity without compromising the capacity to perform arbitrary linear transformations. Despite the observed fluctuations, reinforcement agent implemented by our framework consistently attains the elevated reward values and success rates, even under worst-case scenarios. It should be note that the variability in Fig. S7a-c predominantly stems from the architecture of DQN. As shown in Fig. S7d, transitioning our framework to more stable double DQN architecture yields markedly enhanced training stability. By adopting a more robust double DQN architecture, the fluctuation of the shadow areas of our work in Fig. S7d is much smaller than that of the DQN. It proves that the performance fluctuation is mainly affected by the neural network framework and can be alleviated by more advanced neural network architecture.

Supplementary Note 8: Fidelity of linear transformation and quantified nonlinear fitting capacity of proposed method

We prove the comparative fidelity of linear transformation through modelling a random unitary matrix H by using our proposed method, the data repetition strategy in purely spatial dimension (27), and the phase encoding technique (30). Figures R6a-b illustrate the mean absolute error (MAE) between H and system response H' characterized by these neural networks. The results in Figs. S9a and S9b reveal that, our model retain the arbitrary linear transformation capacity, especially when compared with the model implemented by data repetition strategy in spatial dimension. This outcome further substantiates that our method successfully incorporates nonlinearity without

compromising the capacity to perform arbitrary linear transformations. Figures S9c-f display an estimated system matrix H' of three models alongside the corresponding MAE and spectral norm metrics, evaluated over arbitrary binary input matrix.

We quantify the nonlinearity through cross entropy loss evolution that varies with the network depth. Figures S9g-i plot the accuracy and loss of our model with different model depth for classification task on *MNIST* dataset. As the depth of the network increases, the model's nonlinear fitting capability is progressively enhanced, leading to a continuous improvement in recognition accuracy and a consistent reduction in fitting error. Subsequent declines are attributable to the well-documented degradation phenomena inherent to deep neural networks (i.e., exploding or vanishing gradients) as model depth escalates. These results indicate that, aside from the degradation effects, deeper architectures facilitate better nonlinear fitting capacity.

Supplementary Note 9: Scalability of parallelism through improved asynchronous modulation

We experimentally demonstrate the potential of our framework to be scalable to large-scale parallel applications at two levels: parallel tasks with the same dataset and parallel tasks with the different datasets.

For the parallel task with the same dataset, as shown in Fig. S10a, by using the dataset used in the main text, there are four types of independent labels (i.e., label 1 for gender classification, label 2 for classification of smiling, label 3 for classification of wearing glasses, and label 4 for classification of head pose) and we extend our work to 4 parallel tasks based on single dataset. Training procedure segmented into two distinct phases (i.e., pre-training process and training process) and is demonstrated in **Algorithm 1** below. Weight update conflicts and frequency crosstalk can be alleviated through assistance of information state sensitivity and additional introduction of correction terms in loss function; see details in **Algorithm 1**. Although the length of temporal sequence will affect computing delay to some extent, the influence is limited due to the applications of the frequency multiplexing, especially when compared with existing nonlinear networks. Figures S10b illustrates the accuracy comparison for the four parallel tasks. Our work enables simultaneous multi-task multiplexing that is unattainable by existing optical neural network architectures.

For the parallel tasks involving different datasets like *MNIST* and *Fashion-MNIST* shown in Fig. S10c, we adopt two parallel tasks for the illustration; see the architecture setup and training procedure in **Algorithm 2** below. We use first three metasurface layers for *MNIST* classification and later three

layers for *Fashion-MNIST* classification. Figures R8d and R8e present the accuracy and loss curves for the two parallel classification tasks based on *MNIST* and *Fashion-MNIST* datasets, respectively. Aside from the initial fluctuations observed at the beginning of training, the process progressively stabilizes as the training epochs increase. By increasing the metasurface layers, one can achieve more distinct parallel tasks with different datasets.

Algorithm 1: We employ distributed spatiotemporal metasurfaces to construct a three-layer neural network, with the training procedure being segmented into pre-training process and training process. The pixel size of the metasurfaces is set to $M \times M$, and the temporal sequence length is set to N. In the pre-training phase, parallel task 1 is firstly trained at frequency $f_c + \Delta f_1$ for gender classification. The modulation frequency is set to Δf_1 and the corresponding temporal sequences are $\boldsymbol{S}_{\Delta f_1} = [\boldsymbol{D}, \boldsymbol{W}_1^{\Delta f_1}, \boldsymbol{D}, \boldsymbol{W}_2^{\Delta f_1}, \boldsymbol{D}, \boldsymbol{W}_3^{\Delta f_1}, \boldsymbol{D}, \boldsymbol{W}_4^{\Delta f_1}]$. The information state sensitivity is then measured, and $\frac{M\times M}{4}$ unit cells at the lowest sensitivity are designated as partition 2, of which the modulation frequency is set to $\Delta f_2 = 2\Delta f_1$. The length of temporal sequence at partition 2 is doubled and can be expressed as $\boldsymbol{S}_{\Delta f_2} = [\boldsymbol{D}, \boldsymbol{W}_1^{\Delta f_2}, \boldsymbol{D}, \boldsymbol{W}_2^{\Delta f_2}, \boldsymbol{D}, \boldsymbol{W}_3^{\Delta f_2}, \boldsymbol{D}, \boldsymbol{W}_4^{\Delta f_2}, \boldsymbol{D}, \boldsymbol{W}_5^{\Delta f_2}, \boldsymbol{D}, \boldsymbol{W}_6^{\Delta f_2}, \boldsymbol{D}, \boldsymbol{W}_7^{\Delta f_2}, \boldsymbol{D}, \boldsymbol{W}_8^{\Delta f_2}]$. Subsequently, parallel task 2 is trained at frequency $f_c + \Delta f_2$ for classification of smiling, followed by measurements of information state sensitivity. Again, the $\frac{M\times M}{4}$ unit cells at the lowest sensitivity, excluding those already assigned to partition 2, are selected as partition 3, corresponding to modulation frequency $\Delta f_3 = 4\Delta f_1$, with the temporal sequence being $\boldsymbol{S}_{\Delta f_3} = [\boldsymbol{D}, \boldsymbol{W}_1^{\Delta f_3}, \boldsymbol{D}, \boldsymbol{W}_2^{\Delta f_3}, \ldots, \boldsymbol{D}, \boldsymbol{W}_{16}^{\Delta f_3}]$. The parallel task 3 is trained at $f_c + \Delta f_3$ for classification of wearing glasses. Following analogous procedure, another $\frac{M\times M}{4}$ unit cells are chosen as partition 4, associated with modulation frequency $\Delta f_4 = 8\Delta f_1$, and the temporal sequence becomes $\boldsymbol{S}_{\Delta f_4} = [\boldsymbol{D}, \boldsymbol{W}_1^{\Delta f_4}, \boldsymbol{D}, \boldsymbol{W}_2^{\Delta f_4}, \ldots, \boldsymbol{D}, \boldsymbol{W}_{32}^{\Delta f_4}]$. Parallel task 4 is then trained for classification of head poses. It should note that $\boldsymbol{W}_i^{\Delta f_1} = \boldsymbol{W}_i^{\Delta f_2} = \boldsymbol{W}_i^{\Delta f_3} = \boldsymbol{W}_i^{\Delta f_4}$ for $i = 1,2,3,4$, and $\boldsymbol{W}_j^{\Delta f_2} = \boldsymbol{W}_j^{\Delta f_3} = \boldsymbol{W}_j^{\Delta f_4}$ for $j = 5,6,7,8$, and $\boldsymbol{W}_k^{\Delta f_3} = \boldsymbol{W}_k^{\Delta f_4}$ for $k = 9,10,\ldots,16$. In the training process, the metasurfaces reads all pre-trained weight matrices and employs a four-phase iterative and continuous optimization to alternately update the weight matrices $\boldsymbol{W}^{\Delta f_1}$, $\boldsymbol{W}^{\Delta f_2}$, $\boldsymbol{W}^{\Delta f_3}$, and $\boldsymbol{W}^{\Delta f_4}$. In the first phase, $\boldsymbol{W}^{\Delta f_2}$, $\boldsymbol{W}^{\Delta f_3}$, and $\boldsymbol{W}^{\Delta f_4}$ remain frozen while $\boldsymbol{W}^{\Delta f_1}$ is optimized for parallel task 1. In the second phase, $\boldsymbol{W}^{\Delta f_1}$, $\boldsymbol{W}^{\Delta f_3}$, and $\boldsymbol{W}^{\Delta f_4}$ keep frozen while $\boldsymbol{W}^{\Delta f_2}$ is optimized for parallel task 2. This sequential training approach continues similarly for classification tasks 3 and 4. The four-phase optimization cycle is iterated until convergence is achieved.

Weight update conflicts and frequency crosstalk are mitigated through information state sensitivity and modified loss functions. In the pre-training phase of **Algorithm 1**, the metasurface weight matrices are partitioned into four partitions, each exhibiting reduced channel coupling according to calculated information state sensitivity in Fig.5. Each partition is used to learn independent parallel task. During the training phase, the correction terms are introduced to mitigate the potential accuracy degradation in other parallel tasks. For example, the loss function for parallel task 4 is $\mathcal{L}(\boldsymbol{W}^{\Delta f_4};\boldsymbol{D}) = \mathcal{L}_{CE,4}(\widehat{\boldsymbol{W}^{\Delta f_1}},\widehat{\boldsymbol{W}^{\Delta f_2}},\widehat{\boldsymbol{W}^{\Delta f_3}},\boldsymbol{W}^{\Delta f_4};\boldsymbol{D}) - \lambda_1(\mathbb{E}_{p_{data}(\boldsymbol{D},label1)}[\mathcal{L}_{CE}(\widehat{\boldsymbol{W}^{\Delta f_1}},\widehat{\boldsymbol{W}^{\Delta f_2}},\widehat{\boldsymbol{W}^{\Delta f_3}};\boldsymbol{D})] - \mathbb{E}_{p_{data}(\boldsymbol{D},label1)}[\mathcal{L}_{CE}(\widehat{\boldsymbol{W}^{\Delta f_1}},\widehat{\boldsymbol{W}^{\Delta f_2}},\widehat{\boldsymbol{W}^{\Delta f_3}},\boldsymbol{W}^{\Delta f_4};\boldsymbol{D})]) - \lambda_2(\mathbb{E}_{p_{data}(\boldsymbol{D},label2)}[\mathcal{L}_{CE}(\widehat{\boldsymbol{W}^{\Delta f_1}},\widehat{\boldsymbol{W}^{\Delta f_2}},\widehat{\boldsymbol{W}^{\Delta f_3}};\boldsymbol{D})] - \mathbb{E}_{p_{data}(\boldsymbol{D},label2)}[\mathcal{L}_{CE}(\widehat{\boldsymbol{W}^{\Delta f_1}},\widehat{\boldsymbol{W}^{\Delta f_2}},\widehat{\boldsymbol{W}^{\Delta f_3}},\boldsymbol{W}^{\Delta f_4};\boldsymbol{D})]) - \lambda_3(\mathbb{E}_{p_{data}(\boldsymbol{D},label3)}[\mathcal{L}_{CE}(\widehat{\boldsymbol{W}^{\Delta f_1}},\widehat{\boldsymbol{W}^{\Delta f_2}},\widehat{\boldsymbol{W}^{\Delta f_3}};\boldsymbol{D})] - \mathbb{E}_{p_{data}(\boldsymbol{D},label3)}[\mathcal{L}_{CE}(\widehat{\boldsymbol{W}^{\Delta f_1}},\widehat{\boldsymbol{W}^{\Delta f_2}},\widehat{\boldsymbol{W}^{\Delta f_3}},\boldsymbol{W}^{\Delta f_4};\boldsymbol{D})])$, where $\mathcal{L}_{CE,4}(\widehat{\boldsymbol{W}^{\Delta f_1}},\widehat{\boldsymbol{W}^{\Delta f_2}},\widehat{\boldsymbol{W}^{\Delta f_3}},\boldsymbol{W}^{\Delta f_4};\boldsymbol{D})$ is the cross-entropy function corresponding to network performance at modulation frequency Δf_4 for parallel task 4. $\widehat{\boldsymbol{W}^{\Delta f_1}}$ represents the frozen weight matrices at modulation frequency Δf_1, and $\boldsymbol{W}^{\Delta f_4}$ are the trainable weight matrices at modulation frequency Δf_4. $\mathbb{E}_{p_{data}(\boldsymbol{D},label1)}[\mathcal{L}_{CE}(\widehat{\boldsymbol{W}^{\Delta f_1}},\widehat{\boldsymbol{W}^{\Delta f_2}},\widehat{\boldsymbol{W}^{\Delta f_3}};\boldsymbol{D})] - \mathbb{E}_{p_{data}(\boldsymbol{D},label1)}[\mathcal{L}_{CE}(\widehat{\boldsymbol{W}^{\Delta f_1}},\widehat{\boldsymbol{W}^{\Delta f_2}},\widehat{\boldsymbol{W}^{\Delta f_3}},\boldsymbol{W}^{\Delta f_4};\boldsymbol{D})]$ is the constrain that the network performance at parallel task 1 is supposed to not decreased during the training of parallel task 2. λ is the Lagrange multiplier.

Algorithm 2: The model is constructed utilizing a six-layer metasurfaces architecture. Initially, three 14 × 14 metasurface layers are employed to train the *MNIST* classification task at the frequency $f_c + \Delta f_5$, where modulation frequency of layer 1 to layer 3 is set to Δf_5. Here the modulation frequency of metasurfaces is set to Δf_5, with the corresponding temporal sequence denoted as $S = [\boldsymbol{D}_1^{MNIST},\boldsymbol{W}_1^{\Delta f_5},\boldsymbol{D}_2^{MNIST},\boldsymbol{W}_2^{\Delta f_5},\boldsymbol{D}_3^{MNIST},\boldsymbol{W}_3^{\Delta f_5},\boldsymbol{D}_4^{MNIST},\boldsymbol{W}_4^{\Delta f_5}]$, where input data of *MNIST* dataset is partitioned into 4 feature maps $[\boldsymbol{D}_1^{MNIST},\boldsymbol{D}_2^{MNIST},\boldsymbol{D}_3^{MNIST},\boldsymbol{D}_4^{MNIST}]$. Subsequently, the weight matrices $\boldsymbol{W}^{\Delta f_5}$ are freezed. Concurrently, another three metasurface layers with the size of 16 × 16 are utilized to train the *Fashion-MNIST* classification task at frequency $f_c + \Delta f_6$, wherein the incidence is the output from the third metasurface layer. It should be note that, the input of parallel task 6 is the output of the parallel task 5, i.e., $E_{in} = E_0 G \prod_{l=1}^{3} \boldsymbol{f}_l(\boldsymbol{D},\boldsymbol{W}^{l,\Delta f_5})$. The impact of such input is negligible, as it can be effectively mitigated by normalizing the system response $y = E_{in} G \prod_{l=4}^{6} \boldsymbol{f}_l(\boldsymbol{D},\boldsymbol{W}^{l,\Delta f_6})$ of parallel task 6. The modulation frequency for the layer4 to layer6 is Δf_6, and corresponding temporal sequence is designated as $S = [\boldsymbol{D}_1^{Fashion},\boldsymbol{W}_1^{\Delta f_6},\boldsymbol{D}_2^{Fashion},\boldsymbol{W}_2^{\Delta f_6},\boldsymbol{D}_3^{Fashion},\boldsymbol{W}_3^{\Delta f_6},\boldsymbol{D}_4^{Fashion},\boldsymbol{W}_4^{\Delta f_6}]$, where $[\boldsymbol{D}_1^{Fashion},\boldsymbol{D}_2^{Fashion},\boldsymbol{D}_3^{Fashion},\boldsymbol{D}_4^{Fashion}]$ is the feature map of the input data of the *Fashion-MNIST* dataset. Training process proceeds via a

two-phase iterative optimization scheme: In the first phase, the parameter $\boldsymbol{W}^{\Delta f_6}$ is frozen while $\boldsymbol{W}^{\Delta f_5}$ is optimized for *MNIST* classification; in the second phase, $\boldsymbol{W}^{\Delta f_5}$ is fixed and $\boldsymbol{W}^{\Delta f_6}$ is optimized for *Fashion-MNIST* classification.

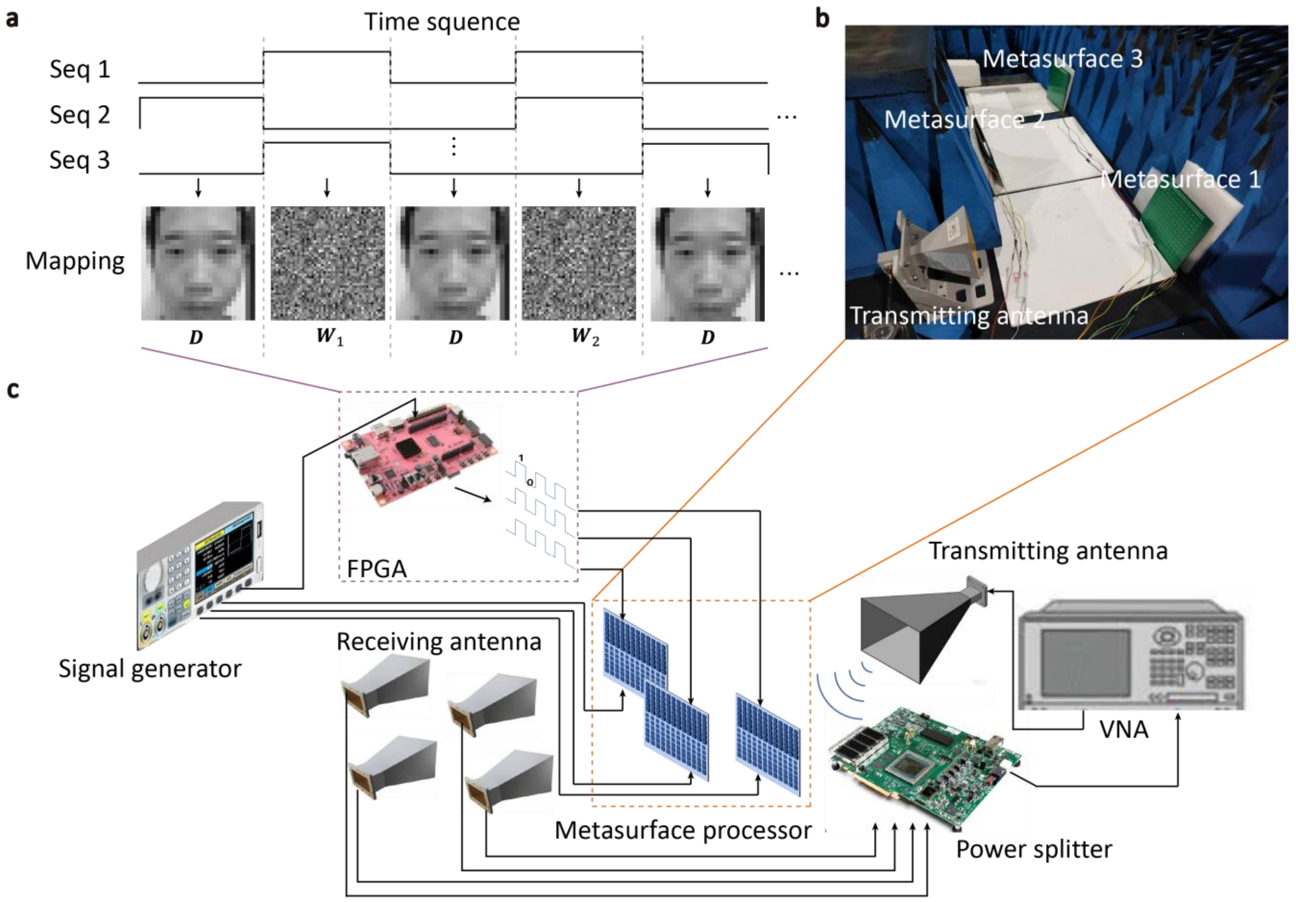

Figure S1 | Experiment details of the nonlinear metasurface processor. a, Setup of time-varying sequence. Each time partition is assigned with a matrix, where the data matrix is assigned to the odd partitions while the weight matrix is assigned to the even partitions. **b**, Experiment scene. The three reflective metasurfaces are placed vertically in a microwave darkroom, and the incident wave is excited by the horn. **c**, Experimental system. The signal generator is used to control the switching of the FPGA and metasurfaces. The power splitter is used to control the measurement of the four ports.

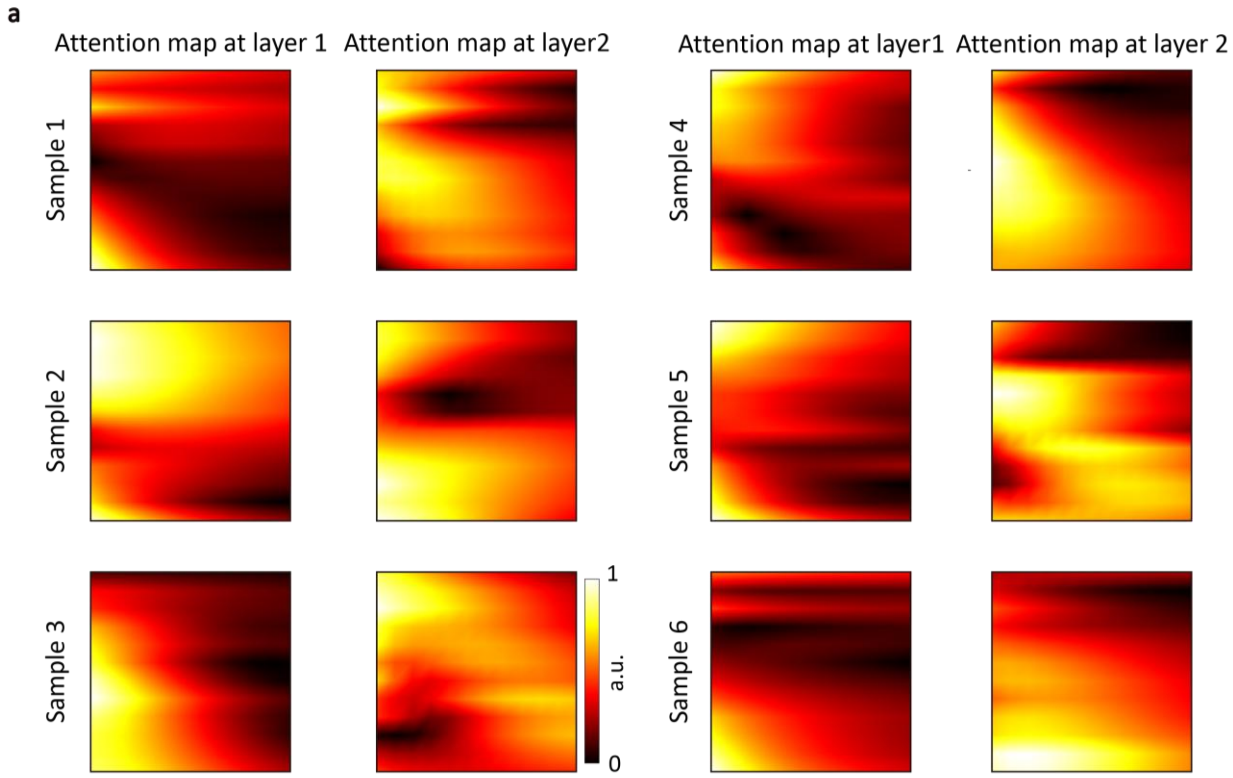

Figure S2 | Comparison of attention map at different layers. a, Attention map calculated by (45) at metasurface layer 1 and metasurface layer 2. The outputs at metasurface layer 2 fully capture the features about gender and head pose of input data, wherein high weight regions are predominantly concentrated at central area and edge of the face.

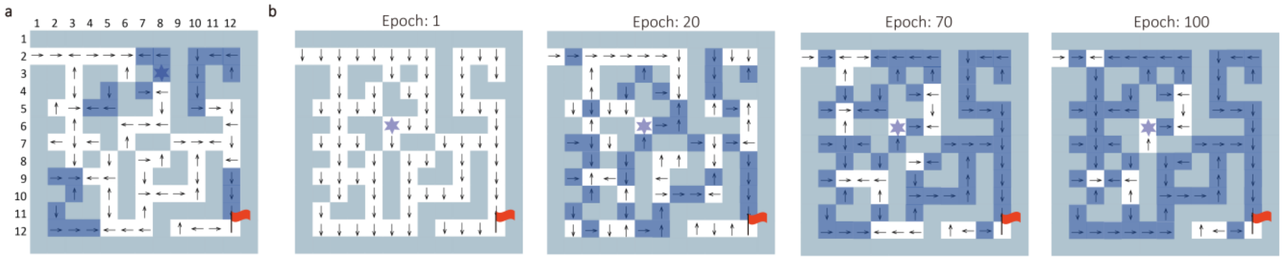

Figure S3 | Policy direction for maze-solving at different training epoch. a, Example of policy control for maze-solving using the proposed untrained metasurface agent. The blue blocks indicate that the metasurface agent remembers the exploration process at this maze location. **b**, Performance of metasurface agent in maze-solving at different training epochs.

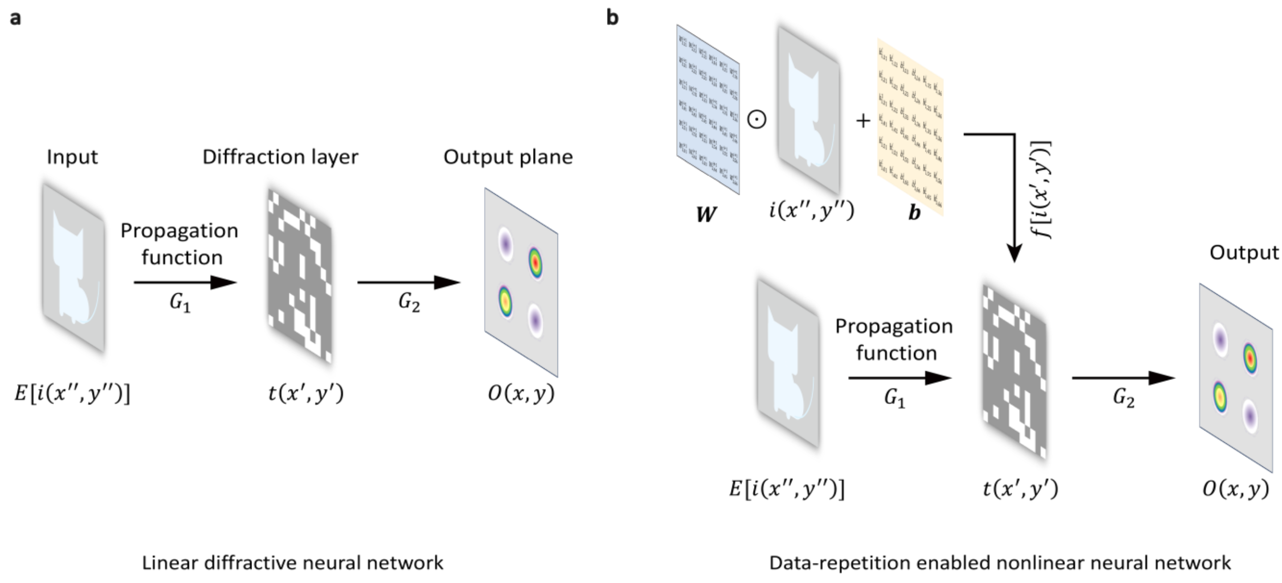

Figure S4 | Architectures of linear diffractive neural network and data-repetition enabled nonlinear diffractive neural network. a, Linear diffractive neural network, which is optimized by diffraction function $t(x', y')$. **b**, Nonlinear diffractive neural network with data-repetition strategy.

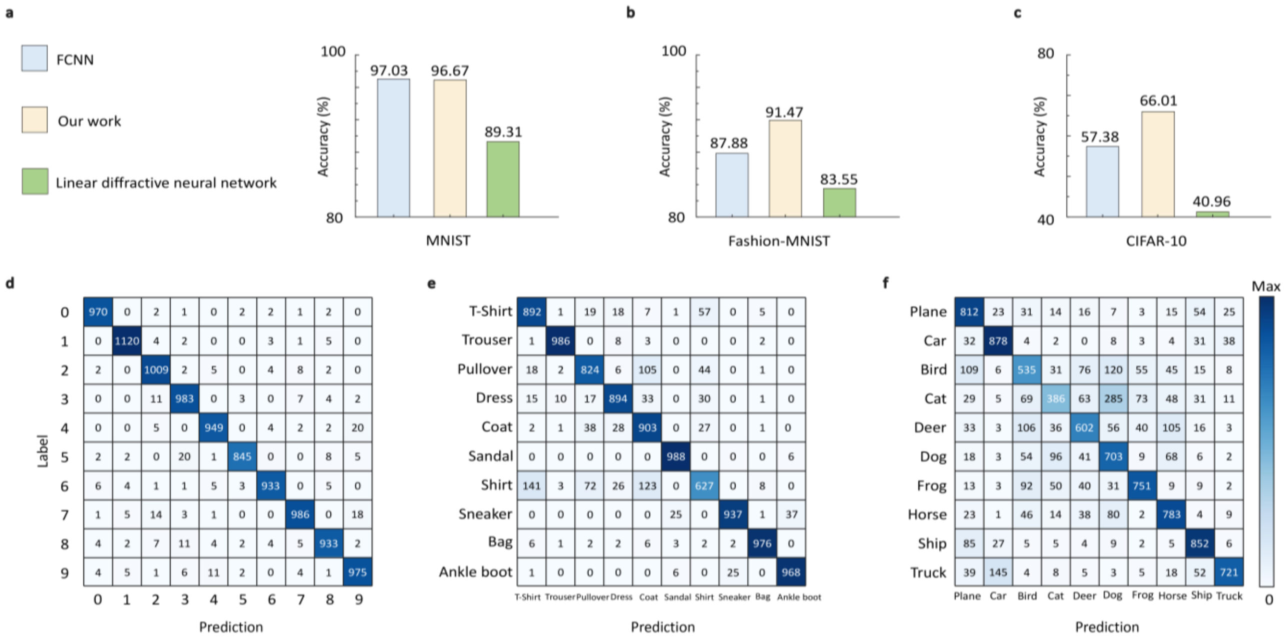

Figure S5 | Performance comparison between fully connected neural network (FCNN), our work, and linear diffractive neural network. Performance comparison of the three methods and the confusion matrices of our framework for (**a**) *MNIST* dataset, (**b**) *Fashion-MNIST* dataset, and (**c**) *CIFAR-10* dataset. **d**, Confusion matrix of our work based on *MNIST* dataset. **e**, Confusion matrix of our work based on *Fashion-MNIST* dataset. **f**, Confusion matrix of our work based on *CIFAR-10* dataset.

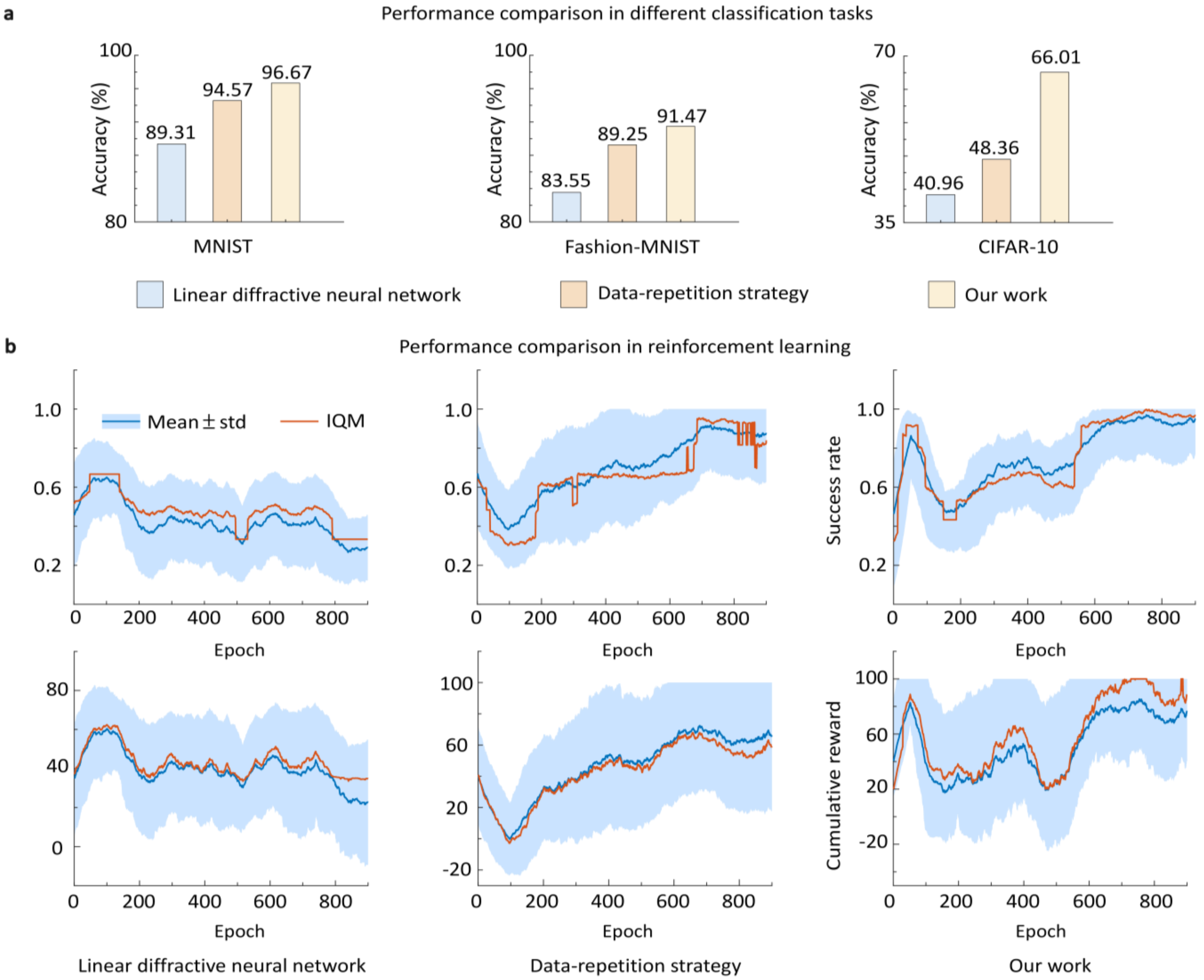

Figure S6 | Comparisons of purely linear system, data-repetition nonlinear strategy, and our method in classification tasks and reinforcement learning based maze-solving. a, Classification accuracy of these models based on different datasets. To make fair comparison without additional variables, all networks are illuminated by unperturbed input data. **b**, Success rates and cumulative returns of the reinforcement agents implemented with our work, data-repetition framework and linear model. In the training process, these models act as the agents to generate the probability of next action for current location in the maze. For a given 11 x 11 maze input, if the car reaches the exit, a reward value of 100 is returned, otherwise a reward value of -1 is returned. The batch size is set to 256 and the models converge within 1,000 epochs. We conducted experiments repeatedly to assess the model's reliability, employing both the standard deviation and interquartile mean (IQM) as quantitative metrics. The extent of the shaded region visually represents the magnitude of variability in the experiment results. The mean represents the expectation of the statistical data and std represents the standard deviation.

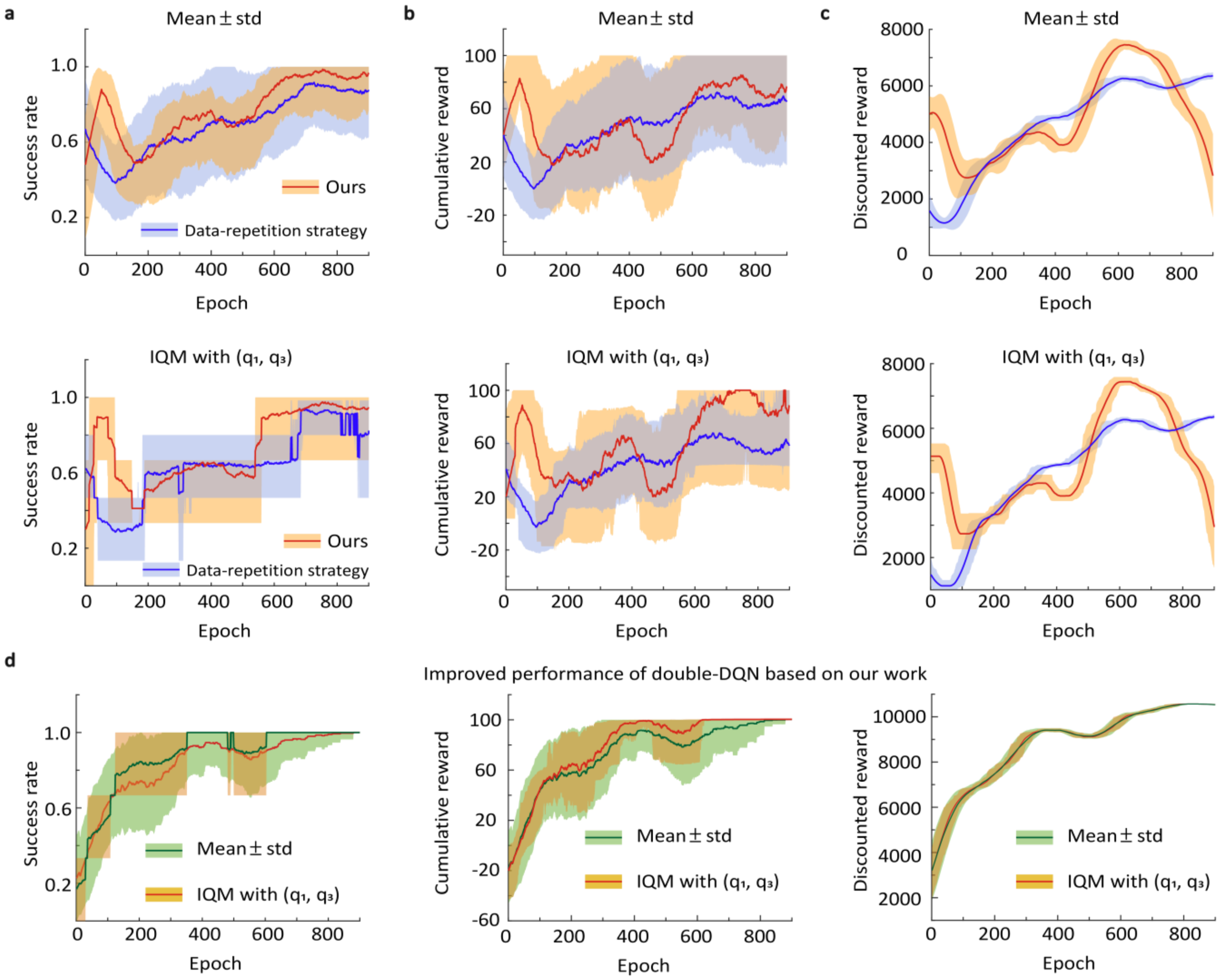

Figure S7 | Evaluation of reinforcement learning for maze-solving by using our framework. The statistical curves are evaluated using sliding window methodology. The standard deviation is represented by blue shaded region and the blue solid lines correspond to the statistical mean of success rates and rewards. The greater of the shaded region, the higher the model variability. Similarly, the red solid line corresponds to the IQM, with the yellow shaded regions bounded by q_1 and q_3, where q_1 encapsulates the lowest 25% and q_3 encapsulates the highest 25% of the statistical distribution. Mean represents the expectation of the statistical data and std represents the standard deviation. The discount factor is set to 0.99. The maximum number of the exploration steps per exploration is set to 200. **a**, Robustness of success rates. **b**, Robustness of cumulative rewards. **c**, Robustness of discounted rewards. **d**, Performance and reliability of double DQN based on our framework. The architecture of the double DQN remains is consistent with that of the DQN in Fig. 6 of the manuscript. The only difference is that action selection is determined by the Q network, while value evaluation is determined by the target Q’ network.

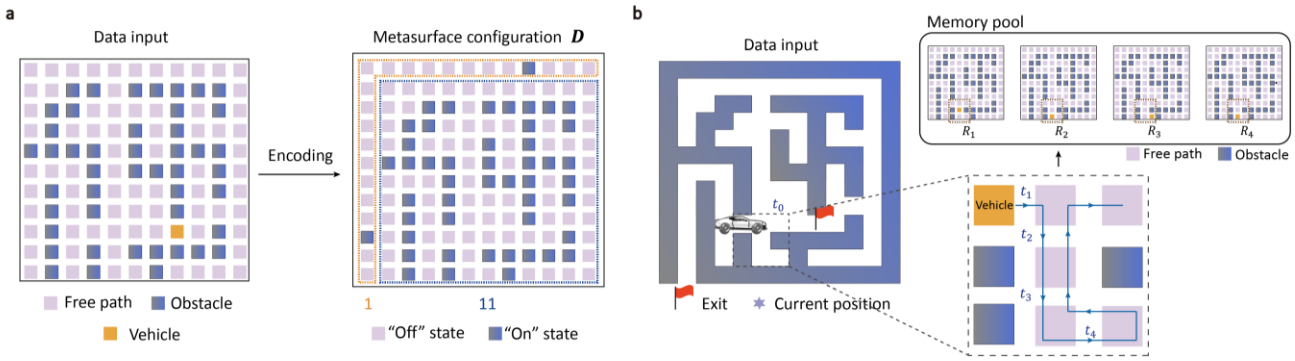

Figure S8 | Data collection and encoding scheme in the maze-solving problem. a, Encoding scheme of 12 x 12 metasurface mapped to the combination of 11 x 11 maze and the location information of the car. **b**, Data collection during the agent's exploration process. At each time step, the car's location at 11x11 maze, and the corresponding reward are recorded and stored in memory pool to build a replay buffer for later training.

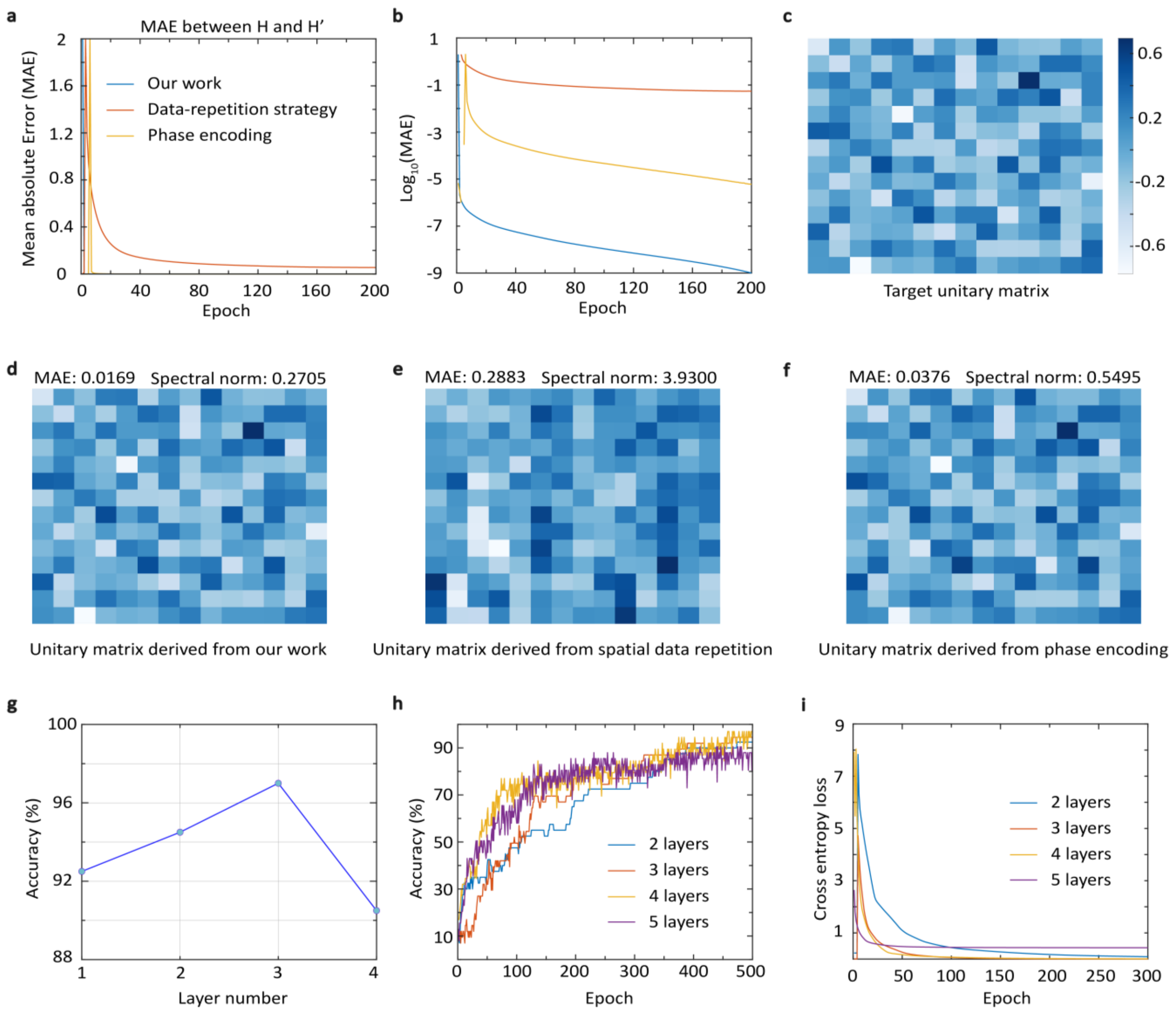

Figure S9 | Nonlinearity quantification of our framework at different model depth and linearity comparison of our framework with existing optical neural network. Performance comparison of modelling unitary matrix H by using our proposed method, the data repetition strategy in purely spatial dimension (27), and the phase encoding technique (30). **a-b**, Mean absolute error curves between H and H' and corresponding logarithm form of models implemented by our framework, data-repetition strategy in purely spatial dimension, and phase encoding. We generated a random 14 × 14 unitary matrix H to serve as the target system’s linear transformation response. The models adopt 3-layers structures and are used to learn H' to fit H. We synthesize the dataset with 11,000 random samples and the dataset is partitioned into 10,000 training samples and 1,000 test samples. **c**, Target system matrix H for linear transformation. **d-f**, System matrices H' derived from trained models and quantitative metrics including mean absolute error and spectral norm. For a random input matrix, output matrices are obtained from the models and the system matrices H' are then

calculated from both input and output matrices of neural networks implemented by our work, data-repetition strategy and phase encoding. **g**, Performance curves of our framework with different model depths. The pixels of metasurface are set to 14 x 14. The length of temporal sequence controlling the metasurface is 8. **h**, Accuracy of our framework across varying network depths. **i**, Cross entropy loss of our framework with different layers.

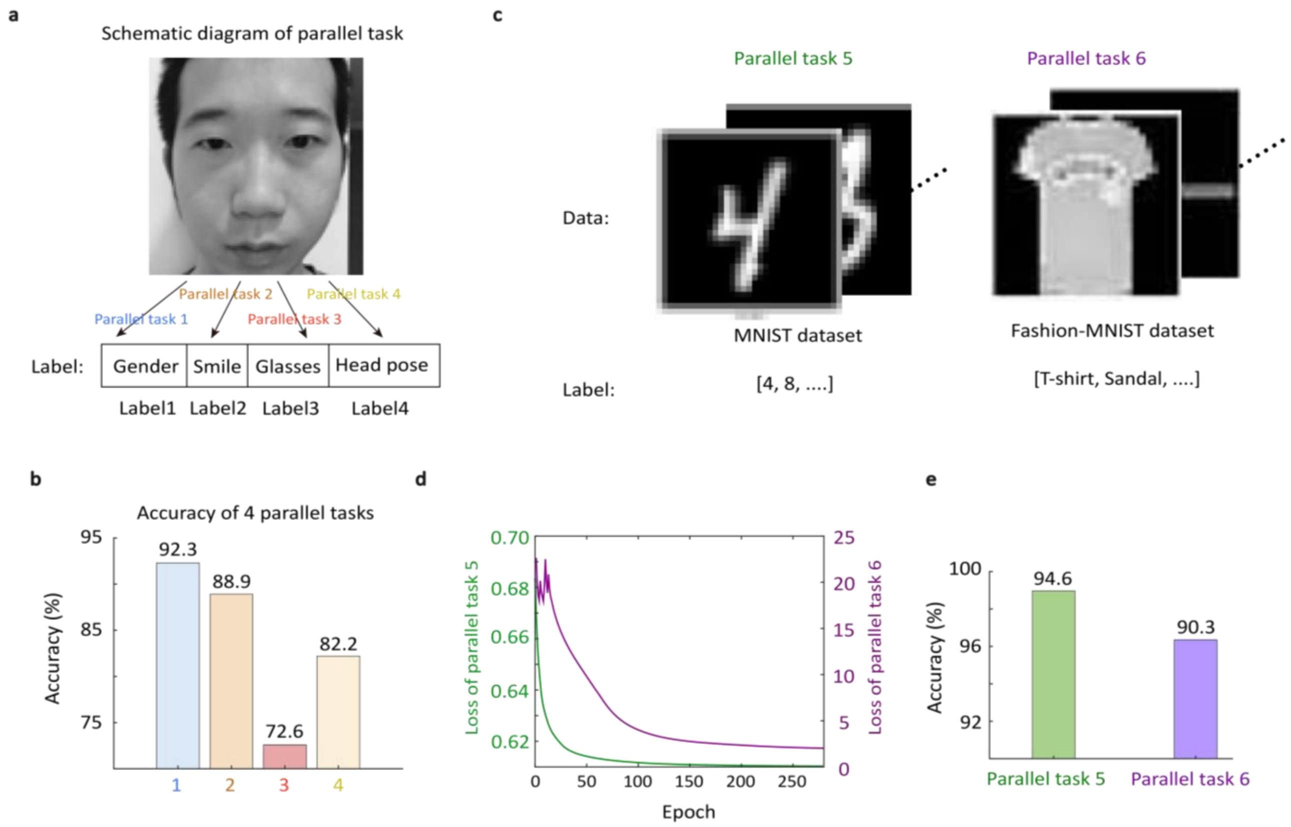

Figure S10 | Scalability to large-scale parallel applications. a, Schematic diagram of data and label for parallel task. Input samples contain the information about gender, smile, glasses, and head pose. Four parallel tasks are conducted by using different labels. **b**, Accuracy of four parallel tasks including classification for gender (i.e., parallel task 1), classification for smiling (i.e., parallel task 2), classification for wearing glasses (i.e., parallel task 3), and classification for head poses (i.e., parallel task 4). **c**, Parallel tasks for different datasets. The dataset of parallel task 5 is *MNIST*, while the dataset of parallel task 6 is *Fashion-MNIST*. **d**, Loss curves of parallel task 5 and parallel task 6. **e**, Accuracy of parallel task 5 and parallel task 6.

REFERENCES

1. Y. LeCun, Y. Bengio, G. Hinton, Deep learning. *Nature* **521**, 436–444 (2015).

2. N. Wu, C. Qian, Z. Wang, X. Zhu, C. Xiao, E. Li, H. Chen, Accelerating cross-scenario metasurface adaptability with plug-and-play kernel. *Adv. Funct. Mater.* **35**, 2502678 (2025).

3. K. He, X. Zhang, S. Ren, J. Sun, "Deep residual learning for image recognition," in *Proceedings of IEEE Conference on Computer Vision and Pattern Recognition* (IEEE, 2016), pp. 770–778.

4. C. Qian, B. Zheng, Y. Shen, L. Jing, E. Li, L. Shen, H. Chen, Deep-learning-enabled self-adaptive microwave cloak without human intervention. *Nat. Photonics* **14**, 383–390 (2020).

5. J. N. P. Martel, L. K. Mueller, S. J. Carey, P. Dudek, G. Wetzstein, Neural sensors: Learning pixel exposures for HDR imaging and video compressive sensing with programmable sensors. *IEEE Trans. Pattern Anal. Mach. Intell.* **42**, 1642–1653 (2020).

6. P. Lin, C. Qian, J. Zhang, J. Chen, X. Zhu, Z. Wang, J. Huang, H. Chen, Enabling intelligent metasurfaces for semi-known input. *Prog. Electromagn. Res.* **178**, 83–91 (2023).

7. Z. Huang, W. Shi, S. Wu, Y. Wang, S. Yang, H. Chen, Pre-sensor computing with compact multilayer optical neural network. *Sci. Adv.* **10**, eado8516 (2024).

8. C. Qian, X. Lin, X. Lin, J. Xu, Y. Sun, E. Li, B. Zhang, H. Chen, Performing optical logic operations by a diffractive neural network. *Light Sci. Appl.* **9**, 59 (2020).

9. T. Yan, J. Wu, T. Zhou, H. Xie, F. Xu, J. Fan, L. Fang, X. Lin, Q. Dai, Fourier-space diffractive deep neural network. *Phys. Rev. Lett.* **123**, 023901 (2019).

10. X. Lin, Y. Rivenson, N. T. Yardimci, M. Veli, Y. Luo, M. Jarrahi, A. Ozcan, All-optical machine learning using diffractive deep neural networks. *Science* **361**, 1004–1008 (2018).

11. G. M. Marega, H. G. Ji, Z. Wang, G. Pasquale, M. Tripathi, A. Radenovic, A. Kis, A large-scale integrated vector–matrix multiplication processor based on monolayer molybdenum disulfide memories. *Nat. Electron.* **6**, 991–998 (2023).

12. Y. Zuo, B. Li, Y. Zhao, Y. Jiang, Y. C. Chen, P. Chen, G. B. Jo, J. Liu, S. Du, All-optical neural network with nonlinear activation functions. *Optica* **6**, 1132–1137 (2019).

13. R. A. Heinz, J. O. Artman, S. H. Lee, Matrix multiplication by optical methods. *Appl. Optics* **9**, 2161–2168 (1970).

14. C. Cuppini, L. Shams, E. Magosso, M. Ursino, A biologically inspired neurocomputational model for audiovisual integration and causal inference. *Eur. J. Neurosci.* **46**, 2481–2498 (2017).

15. F. Ashtiani, A. J. Geers, F. Aflatouni, An on-chip photonic deep neural network for image classification. *Nature* **606**, 501–506 (2022).

16. C. Y. Shen, J. Li, T. Gan, Y. Li, M. Jarrahi, A. Ozcan, All-optical phase conjugation using diffractive wavefront processing. *Nat. Commun.* **15**, 4989 (2024).

17. C. Y. Shen, J. Li, Y. Li, T. Gan, L. Bai, M. Jarrahi, A. Ozcan, Multiplane quantitative phase imaging using a wavelength-multiplexed diffractive optical processor. *Adv. Photonics* **6**, 056003 (2024).

18. Z. Fan, C. Qian, Y. Jia, Z. Wang, Y. Ding, D. Wang, L. Tian, E. Li, T. Cai, B. Zheng, I. Kaminer, H. Chen, Homeostatic neuro-metasurfaces for dynamic wireless channel management. *Sci. Adv.* **8**, eabn7905 (2022).

19. B. Wu, W. Zhang, H. Zhou, J. Dong, D. Huang, P. K. A. Wai, X. Zhang, Chip-to-chip optical multimode communication with universal mode processors. *PhotoniX* **4**, 37 (2023).

20. Z. Xue, T. Zhou, Z. Xu, S. Yu, Q. Dai, L. Fang, Fully forward mode training for optical neural networks. *Nature* **632**, 280–286 (2024).

21. D. Zhang, D. Xu, Y. Li, Y. Luo, J. Hu, J. Zhou, Y. Zhang, B. Zhou, P. Wang, X. Li, B. Bai, H. Ren, L. Wang, A. Zhang, M. Jarrahi, Y. Huang, A. Ozcan, X. Duan, Broadband nonlinear modulation of incoherent light using a transparent optoelectronic neuron array. *Nat. Commun.* **15**, 2433 (2024).

22. C. Qian, Z. Wang, H. Qian, T. Cai, B. Zheng, X. Lin, Y. Shen, I. Kaminer, E. Li, H. Chen, Dynamic recognition and mirage using neuro-metamaterials. *Nat. Commun.* **13**, 2694 (2022).

23. J. Yu, X. Yang, G. Gao, Y. Xiong, Y. Wang, J. Han, Y. Chen, H. Zhang, Q. Sun, Z. Wang, Bioinspired mechano-photonic artificial synapse based on graphene/MoS_2 heterostructure. *Sci. Adv.* **7**, eabd9117 (2021).

24. X. Zhu, C. Qian, E. Li, H. Chen, Negative conductivity induced reconfigurable gain metasurfaces and their nonlinearity. *Phys. Rev. Lett.* **133**, 113801 (2024).

25. A. Baas, J. Karr, H. Eleuch, E. Giacobino, Optical bistability in semiconductor microcavities. *Phys. Rev. A* **69**, 023809 (2004).

26. F. Xia, K. Kim, Y. Eliezer, S. Han, L. Shaughnessy, S. Gigan, H. Cao, Nonlinear optical encoding enabled by recurrent linear scattering. *Nat. Photonics* **18**, 1067–1075 (2024).

27. M. Yildirim, N. U. Dinc, I. Oguz, D. Psaltis, C. Moser, Nonlinear processing with linear optics. *Nat. Photonics* **18**, 1076–1082 (2024).

28. C. C. Wanjura, F. Marquardt, Fully nonlinear neuromorphic computing with linear wave scattering. *Nat. Phys.* **20**, 1434–1440 (2024).

29. W. Nie, Optical nonlinearity-phenomena, applications and materials. *Adv. Mater.* **5**, 520–545 (1993).

30. Y. Li, J. Li, A. Ozcan, Nonlinear encoding in diffractive information processing using linear optical materials. *Light Sci. Appl.* **13**, 173 (2024).

31. C. Qian, Y. Jia, Z. Wang, J. Chen, P. Lin, X. Zhu, E. Li, H. Chen, Autonomous aeroamphibious invisibility cloak with stochastic-evolution learning. *Adv. Photonics* **6**, 016001 (2024).

32. J. Sisler, P. Thureja, M. Y. Grajower, R. Sokhoyan, I. Huang, H. A. Atwater, Electrically tunable space-time metasurfaces at optical frequencies. *Nat. Nanotechnol.* **19**, 1491–1498 (2024).

33. C. Qian, I. Kaminer, H. Chen, A guidance to intelligent metamaterials and metamaterials intelligence. *Nat. Commun.* **16**, 1154 (2025).

34. Y. Jia, C. Qian, Z. Fan, T. Cai, E. Li, H. Chen, A knowledge-inherited learning for intelligent metasurface design and assembly. *Light Sci. Appl.* **12**, 82 (2023).

35. J. W. Liu, G. G. Liu, B. Zhang, Three-dimensional topological photonic crystals. *Prog. Electromagn. Res.* **181**, 99–112 (2024).

36. Z. Fan, Y. Jia, H. Chen, C. Qian, Spatial multiplexing encryption with cascaded metasurfaces. *J. Opt.* **25**, 125105 (2023).

37. D. Bouchet, S. Rotter, A. P. Mosk, Maximum information states for coherent scattering measurements. *Nat. Phys.* **17**, 564–568 (2021).

38. C. Qian, L. Tian, H. Chen, Progress on intelligent metasurfaces for signal relay, transmitter, and processor. *Light Sci. Appl.* **14**, 93 (2025).

39. A. Tennant, B. Chambers, Time-switched array analysis of phase-switched screens. *IEEE Trans. Antennas Propag.* **57**, 808–812 (2009).

40. Z. L. Zhang, M. R. Sabuncu, "Generalized cross entropy loss for training deep neural networks with noisy labels," in *Proceedings of the 32nd International Conference on Neural Information Processing Systems*. (ACM, 2018), pp. 8792–8802.

41. Y. Jia, H. Lu, Z. Fan, B. Wu, F. Qu, M. J. Zhao, C. Qian, H. Chen, High-efficiency Transmissive tunable metasurfaces for binary cascaded diffractive layers. *IEEE Trans. Antennas Propag.* **72**, 4532–4540 (2024).

42. A. M. Shaltout, V. M. Shalaey, M. L. Brongersma, Spatiotemporal light control with active metasurfaces. *Science* **364**, eaat3100 (2019).

43. Z. Zhang, P. Luo, C. C. Loy, X. Tang, "Facial landmark detection by deep multi-task learning," in *Proceeding European on Conference Computer Vision*. (Springer, 2014), pp. 94–108.

44. Z. Zhang, P. Luo, C. C. Loy, X. Tang, Learning deep representation for face alignment with auxiliary attributes. *IEEE Trans. Pattern Anal. Mach. Intell.* **38**, 918–930 (2015).

45. O. Kulce, D. Mengu, Y. Rivenson, A. Ozcan, All-optical information-processing capacity of diffractive surfaces. *Light Sci. Appl.* **10**, 25 (2021).

46. S. Zagoruyko, N. Komodakis, Paying more attention to attention: Improving the performance of convolutional neural networks via attention transfer. arXiv: 1612.03928 [cs.CV] (2016).

47. J. Feldmann, N. Youngblood, M. Karpov, H. Gehring, X. Li, M. Stappers, M. L. Gallo, X. Fu, A. Lukashchuk, A. S. Raja, J. Liu, C. D. Wright, A. Sebastian, T. J. Kippenberg, W. H. P. Pernice, H. Bhaskaran, Parallel convolutional processing using an integrated photonic tensor core. *Nature* **589**, 52–58 (2021).

48. K. Kim, S. Bittner, Y. Zeng, S. Guazzotti, O. Hess, Q. Wang, H. Cao, Massively parallel ultrafast random bit generation with a chip-scale laser. *Science* **371**, 948–952 (2021).

49. S. Wang, M. Chen, J. Ke, Q. Cheng, T. Cui, Asynchronous space-time-coding digital metasurface. *Adv. Sci.* **9**, e2200106 (2022).

50. J. Hüpfl, F. Russo, L. M. Rachbauer, D. Bouchet, J. Lu, U. Kuhl, S. Rotter, Continuity equation for the flow of Fisher information in wave scattering. *Nat. Phys.* **20**, 1294–1299 (2024).

51. G. E. Hinton, P. Dayan, B. J. Frey, R. M. Neal, The “wake-sleep” algorithm for unsupervised neural networks. *Science* **268**, 1158–1161 (1995).

52. Z. Fan, C. Qian, Y. Jia, Y. Feng, H. Qian, E. Li, R. Fleury, H. Chen, Holographic multiplexing metasurface with twisted diffractive neural network. *Nat. Commun.* **15**, 9416 (2024).

53. F. Paredes-Vallés, J. J. Hagenaars, J. Dupeyroux, S. Stroobants, Y. Xu, G. C. H. E. Decroon, Fully neuromorphic vision and control for autonomous drone flight. *Sci. Robot.* **9**, eadi0591 (2024).

54. G. He, C. Qian, Y. Jia, Z. Fan, H. Wang, H. Chen, Twisted metasurfaces for on-demand focusing localization. *Adv. Opt. Mater.* **13**, 2570041 (2024).

55. J. Li, D. Mengu, Y. Luo, Y. Rivenson, A. Ozcan, Class-specific differential detection in diffractive optical neural networks improves inference accuracy. *Adv. Photonics* **1**, 046001 (2019).

56. M. Rahman, J. Li, D. Mengu, Y. Rivenson, A. Ozcan, Ensemble learning of diffractive optical networks. *Light Sci. Appl.* **10**, 1–13 (2021).

57. M. Gu, Q. Zhang, S. Lamon, Nanomaterials for optical data storage. *Nat. Rev. Mater.* **1**, 16070 (2016).

58. J. Deng, Z. Li, J. Li, Z. Zhou, F. Gao, C. Qiu, B. Yan, Metasurface-assisted optical encryption carrying camouflaged information. *Adv. Opt. Mater.* **10**, 2200949 (2022).

59. L. Wang, X. Mao, A. Wang, Y. Wang, Z. Gao, S. Li, L. Yan, Scheme of coherent optical chaos communication. *Opt. Lett.* **45**, 4762–4765 (2020).

60. Y. Song, A. Romero, M. Müller, V. Koltun, D. Scaramuzza, Reaching the limit in autonomous racing: Optimal control versus reinforcement learning. *Sci. Robot.* **8**, eadg1462 (2023).

61. N. Feng, H. Wang, X. Wang, Y. Zhang, C. Qian, Z. Huang, H. Chen, Highly accurate and efficient 3D implementations empowered by deep neural network for 2DLMs-based metamaterials. *Prog. Electromagn. Res.* **180**, 1–11 (2024).

62. A. D. Brink, N. E. Pendock, Minimum cross-entropy threshold selection. *Pattern Recognit.* **29**, 179–188 (1996).

63. L. Zhang, X. Chen, S. Liu, Q. Zhang, J. Zhao, J. Dai, G. Bai, X. Wan, Q. Cheng, G. Castaldi, V. Galdi, T. Cui, Space-time-coding digital metasurfaces. *Nat. Commun.* **9**, 4334 (2018).

64. O. Kulce, D. Mengu, Y. Rivenson, A. Ozcan, All-optical synthesis of an arbitrary linear transformation using diffractive surfaces. *Light Sci. Appl.* **10**, 196 (2021).

65. Y. Xu, Z. Liu, M. Tegmark, T. S. Jaakkola, Poisson flow generative models. *Adv. Neural Inf. Process. Syst.* **35**, 16782–16795 (2022).

66. J. Ho, A. Jain, P. Abbeel, Denoising diffusion probabilistic models. *Adv. Neural Inf. Process. Syst.* **33**, 6840–6851 (2020).
